# Management of bile acid diarrhea in Italy: a survey

**DOI:** 10.1007/s11739-025-04060-9

**Published:** 2025-09-12

**Authors:** Giovanni Marasco, Giovanni Barbara, Massimo Bellini, Piero Portincasa, Vincenzo Stanghellini, Bruno Annibale, Antonio Benedetti, Giovanni Cammarota, Walter Fries, Paolo Usai Satta, Enrico Stefano Corazziari

**Affiliations:** 1 IRCCS Azienda Ospedaliero Universitaria Di Bologna, Bologna, Italy; 2 Department of Medical and Surgical Sciences, University of Bologna, Bologna, Italy; 3 Gastrointestinal Unit, Department of Translational Research and New Technologies in Medicine and Surgery, University of Pisa, Pisa, Italy; 4 Clinica Medica “A. Murri”, Department of Precision and Regenerative Medicine and Ionian Area (DiMePre-J), University of Bari Aldo Moro, Bari, Italy; 5 Department of Medical-Surgical Sciences and Translational Medicine, Sant’Andrea Hospital, Sapienza University of Rome, Rome, Italy; 6 Clinic of Gastroenterology and Hepatology, Università Politecnica Delle Marche, Ospedali Riuniti-University Hospital, Ancona, Italy; 7 Gastroenterology Unit, Fondazione Policlinico A Gemelli IRCCS, Catholic University of Medicine, Rome, Italy; 8 Clinical Unit of Gastroenterology, Department of Clinical and Experimental Medicine, University of Messina, Messina, Italy; 9 Gastroenterology Unit, ARNAS G. Brotzu, Cagliari, Italy; 10 Department of Gastroenterology, IRCCS Humanitas Research Hospital, Rozzano, Milano Italy

**Keywords:** Bile acid diarrhea, Cholestyramine, Colesevelam, Diarrhea, Irritable bowel syndrome, Survey

## Abstract

Bile acid diarrhea (BAD) is a common, under-investigated cause of chronic diarrhea. We aimed to assess the current management of BAD among a group of Italian physicians. A survey was developed by a task force of experts and distributed via the Internet to Italian physicians members of the main Italian gastroenterological associations. Ninety-four physicians accepted to participate, of whom 44% were females. The majority of participants were gastroenterologists (63%) and the mean age was 50.5 years. No differences in the rate of BAD diagnosis among patients with chronic diarrhea were found according to medical specialization. Gastroenterologists reported a higher prevalence of BAD compared with other physicians/general practitioners (1% vs 0.3%). BAD suspicion is mostly raised in the presence of watery stools and > 3 bowel movements/day and the exclusion of organic/drug-related diseases. BAD diagnosis was assessed with 75SeHCAT (67.8% of gastroenterologists and 51.4% of other physicians), followed by a trial of cholestyramine (30.5% of gastroenterologists and 31.4% of other physicians). Therapies most prescribed for BAD were cholestyramine, a low-fat diet, and stool thickeners. BAD is a common condition generally suspected in the presence of chronic watery diarrhea. 75SeHCAT availability influences the awareness of this disease. Therapies currently are often not able to guarantee adequate symptom relief.

## Introduction

Bile acid malabsorption (BAD) is a common under-investigated cause of chronic diarrhea (25–33%) with a prevalence of about 1% in the general population [1–3]. About 25–33% of unexplained chronic diarrhea is due to BAD and it is also present in about one-third of patients with irritable bowel syndrome diarrhea-predominant (IBS-D) [4–6]. BAD results from dysregulation of the enterohepatic recirculation of bile acids (BAs) and its consequent alteration of bile acid production driven by several mechanisms [7]. It finally hesitates in an excessive level of BAs in the lower gastrointestinal tract, which in turn can stimulate water and sodium excessive transport, mucosal damage, mucus secretion, increased lower gastrointestinal motility, and stimulation of defecation [8–10], finally leading to BA-associated diarrhea (BAD). Therefore, patients with BAD complain of increased frequency of watery chronic diarrhea, fecal urgency, abdominal pain, bloating, and fecal incontinence [11]. Affected individuals may also report systemic symptoms including fatigue, dizziness and feeling of fainting [12]*.* Currently, the gold standard diagnostic method for identifying BAD and assessing its degree of severity is the 75-Selenium-HomotauroCholic Acid Test (75SeHCAT), consisting of a radio-labelled synthetic conjugated bile acid (23-seleno-25-homo tauro-cholic acid) that is orally administered, secreted in bile and then reabsorbed in the terminal ileum [13]. According to the retention of this radio-labeled marker it is possible to ascertain the presence and severity of BAD, which is directly correlated to the response to bile acid sequestrants [14]. Other diagnostic markers have been recently proposed, such as 7αC4 or FGF19 [15–18]. However, also due to the insufficient availability of diagnostic tests and the lack of awareness among physicians on this condition, BAD is often overlooked and misdiagnosed, leading to delayed diagnosis up to 5 years after symptom onset [14]. The diagnostic delay impacts the quality of life of affected individuals and increases the healthcare burden with unnecessary diagnostic tests [19]. Current treatment for these patients includes dietary modification and the use of bile acid sequestrants (BASTs, e.g. cholestyramine, colesevelam, colestipol) [7]. However, BASTs are often ineffective when used in uninvestigated patients or poorly tolerated due to side effects such as constipation, abdominal pain, bloating, fullness, nausea, and flatulence [20], and the poor palatability of cholestyramine and colestipol, which result in low compliance.

We aimed to assess the current knowledge and management of BAD among a group of Italian physicians in order to increase awareness of this condition and target possible knowledge gaps in the near future within this field.

## Methods

A survey investigating BAD knowledge and management in Italy was developed by a task force of experts (GB, MB, VS, ESC).

The survey was developed in Italian and consisted of 22 questions, including different areas of interest: (a) demographics and work position, (b) knowledge of BAD epidemiology and management, and (c) personal clinical experience of BAD. Survey questions and responses are available in Tables 1 and 2. The electronic survey was distributed via Internet to Italian physicians with an interest in gastroenterology and to members of the main Italian gastroenterological associations between May and September 2022. A secured online survey was provided by a professional company (Qualtrics, LLC, Provo, UT). The survey was anonymous and had built-in quality-assurance measures to exclude poor-quality responders. The software ensured that there were no missing answers to compulsory questions and had automated skip patterns, resulting in complete and accurate symptom pattern information.

**Table 1:** Response to survey questions according to medical specialization

	Gastroenterologist *N* (%) or mean (SD) *n* = 59	Other physicians *N* (%) or mean (SD) *n* = 35	*P*
Sex (Female)	22 (37.2)	19 (54.3)	0.108
Age	54.5 (15.5)	43.4 (15.7)	< 0.001
Geographical area			
Northern Italy	29 (49.2)	16 (45.7)	0.747
Central Italy	16 (27.1)	8 (22.9)	0.647
Southern/Islands Italy	14 (23.7)	11 (31.4)	0.414
Rate of patients with BAD in the general population			
0.3%	16 (27.1)	17 (48.6)	0.035
0.05%	7 (11.9)	3 (8.6)	0.617
1%	24 (40.7)	7 (20)	0.039
10%	4 (6.8)	0 (0)	0.293
I don’t know	8 (13.6)	8 (22.9)	0.246
Number of patients/year with chronic diarrhea	149.8 (188.3)	77.8 (106.3)	0.002
Rate of patients among those with chronic diarrhea with BAD	12.6 (21.3)	7.2 (9.1)	0.205
Clinical suspicion of BAD			
Watery stools	0	1 (2.9)	0.372
Watery stools and > 3 CSBM/day	1 (1.7)	1 (2.9)	0.706
Watery stools and exclusion of organic/drug-related diseases	4 (6.8)	6 (17.1)	0.115
Watery stools and > 3 CSBM/day and exclusion of organic/drug-related diseases	36 (61)	23 (65.7)	0.649
Watery stools and > 3 CSBM/day not responsive to previous therapies	18 (30.5)	4(11.4)	0.035
Diagnostic evaluation for BAD			
Colonoscopy and biopsies	0	4 (11.4)	0.017
Cholestyramine therapy response	18 (30.5)	11 (31.4)	0.926
Budesonide therapy response	0	0	1
75SeHCAT	40 (67.8)	18 (51.4)	0.115
Other	1 (1.7)	2 (5.7)	0.283
Dose of cholestyramine used for ex-adiuvantibus diagnosis			
2 g/die	3 (16.7)	2 (18.2)	0.917
4 g/die	5 (27.8)	7 (63.6)	0.057
6 g/die	1 (5.6)	1 (9.1)	0.715
8 g/die	7 (38.9)	1 (9.1)	0.082
Other	2 (11.1)	0	0.512
Duration of cholestyramine therapy for ex-adiuvantibus diagnosis (days)			
3	0	0	1
7	2 (11.1)	5 (45.5)	0.036
10	3 (16.7)	3 (27.3)	0.494
14	4 (22.2)	2 (18.2)	0.794
28	9 (50)	1 (9.1)	0.041
Belief that 75SeHCAT diagnosis is better than empiric therapy			0.293
Yes	26 (44.1)	17 (50)	
No	17 (28.8)	5 (14.7)	
I don’t know	16 (27.1)	12 (35.3)	
Perceived diagnostic accuracy of 75SeHCAT for BAD diagnosis			
20–30%	1 (1.7)	2 (5.9)	0.284
60–70%	25 (42.4)	15 (44.1)	0.963
90–100%	26 (44.1)	9 (26.5)	0.075
I don’t know	7 (11.9)	8 (23.5)	0.160
75SeHCAT prescription in clinical activity (if ever requested)	22 (37.3)	9 (26.5)	0.287
Regional availability of 75SeHCAT			0.004
Yes	26 (46.4)	12 (37.5)	
No	14 (25)	1 (3.1)	
I don’t know	16 (28.6)	19 (59.4)	
Number of 75SeHCAT prescribed in the last year	3.2 (20)	2.8 (9.1)	0.213
Belief that 75SeHCAT test is enough for BAD diagnosis			0.059
Yes	41 (73.2)	16 (50)	
No	7 (12.5)	5 (15.6)	
I don’t know	8 (14.3)	11 (34.4)	
Belief that 7αC4 or FGF19 are enough accurate for BAD diagnosis			0.644
Yes	11 (19.6)	6 (18.7)	
No	17 (30.4)	7 (21.9)	
I don’t know	28 (50)	19 (59.4)	
Satisfied by therapies available for BAD	19 (33.9)	10 (31.3)	0.797
Rate of patients with BAD with satisfying symptom control and quality of life	54.7 (26.2)	42.4 (30)	0.070
Satisfied about own knowledge of BAD	14 (25)	6 (18.8)	0.501
Belief in the usefulness of updates on BAD	55 (98.2)	32 (100)	0.447

**Table 2:** Response to survey questions according to 75SeHCAT regional availability

	No 75SeHCAT availability *N* (%) or mean (SD) *n* = 56	75SeHCAT availability *N* (%) or mean (SD) *n* = 38	*P*
Sex (Female)	25 (44.6)	16 (42.1)	0.808
Age	50.6 (15.7)	50.3 (17.6)	0.896
Geographical area			
Northern Italy	24 (42.9)	21 (55.3)	0.237
Central Italy	14 (25)	10 (26.3)	0.886
Southern/Islands Italy	18 (32.1)	7 (18.4)	0.140
Rate of patients with BAD in the general population			
0.3%	17 (30.4)	16 (42.1)	0.097
0.05%	8 (14.3)	2 (5.3)	0.197
1%	16 (28.6)	15 (39.5)	0.270
10%	1 (1.8)	3 (7.9)	0.150
I don’t know	14 (25)	2 (5.3)	0.124
Number of patients/year with chronic diarrhea	102.8 (110.5)	154.9 (224.2)	0.272
Rate of patients among those with chronic diarrhea with BAD	9.3 (13.6)	12.6 (23.1)	0.666
Clinical suspicion of BAD			
Watery stools	0	1 (2.6)	0.409
Watery stools and > 3 CSBM/day	2 (3.6)	0	0.513
Watery stools and exclusion of organic/drug-related diseases	5 (8.9)	5 (13.2)	0.514
Watery stools and > 3 CSBM/day and exclusion of organic/drug-related diseases	35 (62.5)	24 (63.2)	0.948
Watery stools and > 3 CSBM/day not responsive to previous therapies	14 (25)	8 (21.1)	0.657
Key diagnostic evaluation for BAD			
Colonoscopy and biopsies	3 (5.4)	1 (2.6)	0.521
Cholestyramine therapy response	24 (42.9)	5 (13.2)	0.002
Budesonide therapy response	0	0	1
75SeHCAT	26 (46.4)	32 (84.2)	< 0.001
Other	3 (5.4)	0	0.270
Dose of cholestyramine used for ex-adiuvantibus diagnosis (in cholestyramine users)			
2 g/die	4 (16.7)	1 (20)	0.857
4 g/die	11 (45.8)	1 (20)	0.286
6 g/die	2 (8.3)	0	1
8 g/die	6 (25)	2 (40)	0.495
Other	1 (4.2)	1 (20)	0.204
Duration of cholestyramine therapy for ex-adiuvantibus diagnosis (in cholestyramine users), days			
3	0	0	1
7	6 (25)	1 (20)	0.812
10	6 (25)	0	0.553
14	5 (20.8)	1 (20)	0.967
28	7 (29.2)	3 (60)	0.187
Belief that 75SeHCAT diagnosis is better than empiric therapy			0.977
Yes	25 (45.5)	18 (47.4)	
No	13 (23.6)	9 (23.7)	
I don’t know	17 (30.9)	11 (28.9)	
Perceived diagnostic accuracy of 75SeHCAT for BAD diagnosis			
20–30%	2 (3.6)	1 (2.6)	0.799
60–70%	20 (36.4)	20 (52.6)	0.104
90–100%	20 (36.4)	15 (39.5)	0.711
I don’t know	13 (23.6)	2 (5.3)	0.020
75SeHCAT prescription in clinical activity (if ever requested)	6 (10.9)	25 (65.8)	< 0.001
Number of 75SeHCAT prescribed in the last year	0.5 (2.2)	6.5 (25.2)	< 0.001
Belief that 75SeHCAT test is enough for BAD diagnosis			0.004
Yes	31 (62)	26 (68.4)	
No	3 (6)	9 (23.7)	
I don’t know	16 (32)	3 (7.9)	
Belief that 7αC4 or FGF19 are enough accurate for BAD diagnosis			0.081
Yes	11 (22)	6 (15.8)	
No	9 (18)	15 (39.5)	
I don’t know	30 (60)	17 (44.7)	
Satisfied by therapies available for BAD	16 (32)	13 (34.2)	0.827
Rate of patients with BAD with satisfying symptom control and quality of life	46.2 (29.3)	56.5 (25.4)	0.144
Satisfied about own knowledge of BAD	4 (8)	16 (42.1)	< 0.001
Belief in the usefulness of updates on BAD	49 (98)	38 (100)	0.381

### Statistical analysis

Data are presented as counts and percentages for the categorical variables and mean and standard deviation (SD) for the continuous variables. The categorical variables were compared using the Chi-squared or Fisher’s exact tests as appropriate. For multiple categorical variables, the Chi-squared test of independence was used. The continuous variables were compared using the t-test or the Kruskal–Wallis test as appropriate. The differences in responses between gastroenterologists and other physicians, as well as between physicians with and without regional availability of 75SeHCAT were calculated. The probability values were two-sided; a probability value of less than 0.05 was considered statistically significant. Statistical analysis was performed with STATA 17.0 (SE, Standard Edition, College Station, TX: StataCorp LP).

## Results

### Participants’ demographics and clinical experience

Ninety-four participated in the survey, of whom 47 were females (44%). The majority of participants were gastroenterologists (63%), while the remaining 37% included internal medicine physicians (12.8%), general surgeons (3%), general practitioners (9.6%) and other healthcare professionals (11.6%).

Tables 1 and 2 report survey question responses according to medical specialization and 75SeHCAT availability, respectively. The mean age of participants was 50.5 years (SD 16.4), with a significantly older age in the group of gastroenterologists (54.5 years) compared to other physicians (43.4 years) (*p* < 0.001). No differences were found according to 75SeHCAT availability. About half of the participants (48%) were from Northern Italy, 26% from Central and 26% from Southern Italy. No differences in geographical areas of origin were found according to medical specialization or 75SeHCAT availability.

Gastroenterologists reported to visit a significantly higher number of patients with chronic diarrhea when compared to other physicians (149.8 vs 77.8, *p* = 0.002). However, no differences in the rate of BAD diagnosis within patients with chronic diarrhea were found among the two groups. Different estimations of BAD prevalence in the general population were reported by the two groups since gastroenterologists mainly reported a prevalence of about 1% (40.7% of gastroenterologists), while other physicians mainly reported an estimated prevalence of 0.3% (48.6% within this group). No differences in the number of visits for chronic diarrhea and BAD estimation were found according to 75SeHCAT availability.

### BAD diagnosis

The presence of watery stools, > 3 complete spontaneous bowel movement (CSBM)/day and the exclusion of organic/drug-related diseases were identified as a criterion for BAD diagnosis by most respondents to the survey, without differences among groups except for about one-third of gastroenterologists which additionally reported to suspect BAD in case of failure of previous therapies for chronic diarrhea. BAD diagnosis was generally reported to be assessed by 75SeHCAT (67.8% of gastroenterologists and 51.4% of other physicians; *p* = 0.115), followed by the assessment of the clinical response to a trial of cholestyramine (30.5% of gastroenterologists and 31.4% of other physicians; *p* = 0.926). As expected, 75SeHCAT was the most prescribed test, based on regional availability (84.2% when 75SeHCAT available vs. 46.4% when unavailable, *p* < 0.001). The cholestyramine trial test was prescribed by 42.9% of physicians without 75SeHCAT regional availability.

Among the 29 participants reporting adopting ex adjuvantibus diagnostic criteria based on cholestyramine therapy, no differences were found between gastroenterologists and other physicians, nor according to 75SeHCAT availability in terms of doses employed. Overall, 17% of participants reported using 2 g/die, 41.4% of participants 4 g/die, 6.9% of participants 6 g/die, 27.6% of participants 8 g/die and 6.9% other amounts. When the duration of the cholestyramine trial for ex adjuvantibus diagnosis was considered, 24.4% of participants reported a trial of 7 days, 20.7% of 10 days, 20.7% of 14 days and 34.5% of 28 days. About half of the other physicians adopted a 7-days trial, while half of the gastroenterologists 28 days. No differences were found according to 75SeHCAT availability.

Only about half of the participants believed that a BAD diagnosis using the 75SeHCAT test was more accurate than using an ex adjuvantibus diagnosis with a cholestyramine trial, without differences between groups.

Overall, one-third of participants reported having prescribed 75SeHCAT in their routine clinical practice.

No differences in the number of participants prescribing 75SeHCAT and in the number of 75SeHCAT prescribed were found according to medical specialization, while huge differences were found according to 75SeHCAT availability (65.8% of prescribers when locally available vs 10.9% when unavailable, *p* < 0.001 and a mean of 6.5 75SeHCAT/per year prescribed when available vs 0.5 when unavailable, *p* < 0.001).

**Fig. 1:**
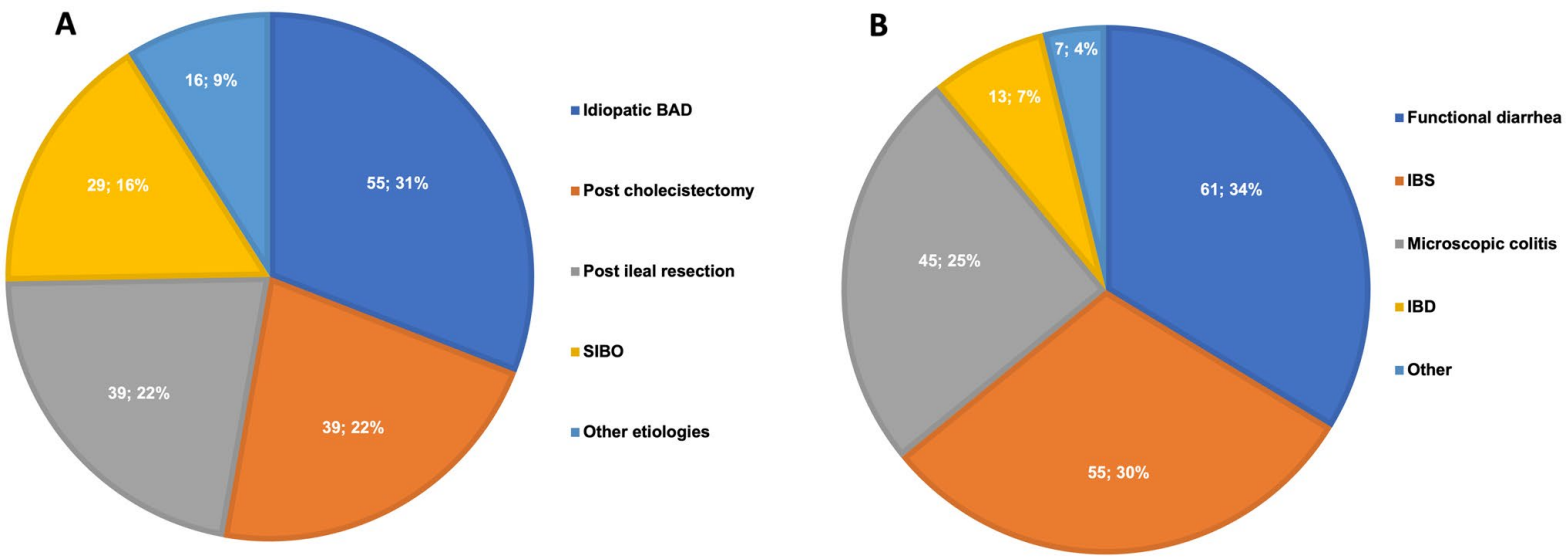
Diagnosis after 75SeHCAT testing in patients with chronic unexplained diarrhea reported by respondents in their own experience: **A** BAD subtypes (%) and **B** other diagnosis after BAD exclusion (%) (multiple answers were allowed). *Abbreviations: BAD, bile acid malabsorption; SIBO, small intestinal bacterial overgrowth; IBS, irritable bowel syndrome; IBD, inflammatory bowel diseases*

When BAD was confirmed by 75SeHCAT the most frequent diagnoses were idiopathic BAD in the majority of cases, followed by post-surgical BAD (both post-cholecystectomy and post-ileal resection), small intestinal bacterial overgrowth, and others (Fig. 1A). When BAD was not confirmed by 75SeHCAT, the final diagnoses causing chronic diarrhea were functional diarrhea, irritable bowel syndrome, microscopic colitis and inflammatory bowel diseases in decreasing order of reported occurrence (Fig. 1B).

Only about 20% of respondents supported the accuracy of 7αC4 or FGF19 for BAD diagnosis, without differences between groups.

### BAD management

**Fig. 2:**
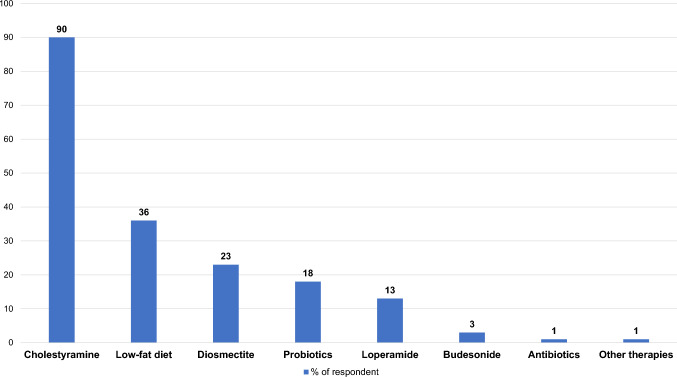
Therapies most commonly prescribed for BAD reported by participants in the survey (multiple answers were allowed)

Overall, 33% of participants reported being satisfied with the currently available therapies for BAD, without differences between groups. The most commonly prescribed therapies were cholestyramine, a low-fat diet, stool thickeners (i.e. diosmectite), and loperamide (Fig. 2). About half of the respondents from both groups reported to have achieved symptom control in less than 50% of patients. The overall satisfaction about the personal knowledge on BAD was 23%, with a significantly different rate of up to 42% of respondents with 75SeHCAT availability (*p* < 0.001). Almost all participants claimed the need for updates on BAD.

## Discussion

The present survey investigated the knowledge and clinical attitudes of a group of Italian physicians in the management of BAD aiming to highlight the current clinical practice and guide future updates on this topic to increase awareness of this condition. To the best of our knowledge, only a previous survey was published on this topic [14]. It was carried out in the UK, including a small group of selected experts in this field (*n* = 21) and it was focused on improving diagnostic rates and management of BAD through a consensus on specific clinical cases. Therefore, the results are not fully comparable to our experience.

In our study we included a heterogeneous sample of physician and compared the answers from gastroenterologist to those of the remaining participant in the survey, which were mainly internal medicine doctors or surgeons. These medical specializations represent the three major types of physicians possibly facing this disease in their clinical practice. Although about half of the participants were from Northern Italy, where there is the greatest availability of 75SeHCAT test in Italy, we surprisingly found no differences in 75SeHCAT availability among the two groups.

A considerable lack of knowledge was found among gastroenterologists and other physicians with an interest in digestive diseases. Only one-third of respondents correctly estimate the actual burden of BAD. Since the prevalence of chronic diarrhea in the general population has been estimated at around 5% and BAD is responsible for 26–50% of these cases [2, 21], BAD prevalence can be estimated to affect around 1% of the general population. When patients with IBS-D are considered, this rate increases up to 28.1%, according to a meta-analysis including more than 900 patients [4]. Therefore, about half of our respondents underestimated the burden of BAD. Surprisingly, despite the higher number of visits made by gastroenterologists for patients with chronic diarrhea, the rate of BAD diagnosis was comparable between gastroenterologists and other physicians. This can be explained by a selection bias of participants with a specific interest in these topics. The most commonly reported clinical criteria for BAD diagnosis were the presence of watery stools, > 3 CSBM/day, and the exclusion of organic/drug-related diseases. This finding is in keeping with what was reported in the survey by Walters et al. since the experts reported loose stools and frequency greater than 3 times/day as common findings [14]. However, the recently published Canadian guidelines for BAD management [16] suggested against using symptom presentation for the initial assessment to identify patients with possible BAD, while recommending using risk factors (history of terminal ileal resection, cholecystectomy, or abdominal radiotherapy) for the initial assessment of patients with chronic non-bloody diarrhea. Most of the participants in our survey reported using 75SeHCAT as a gold standard method for BAD diagnosis, when available, in line with international guidelines [6, 16]. Our data underline that the prescription of 75SeHCAT in clinical practice, the perceived accuracy of this test, and the general awareness and knowledge of BAD are directly correlated with 75SeHCAT test availability in the place of origin. On this line and supporting the importance of 75SeHCAT availability, we found no difference in the number of 75SeHCAT prescribed by gastroenterologists and other physicians operating in the same area.

The variation in 75SeHCAT utilization among physicians likely reflects differences in local test availability, institutional protocols, and physician familiarity with BAD. In regions where the test is not accessible, clinicians may rely more on empirical therapy or alternative diagnostic approaches, leading to inconsistent practices [1]. This inconsistency underscores the need for standardized diagnostic pathways and wider dissemination of guideline-based approaches [14].

When the response to a cholestyramine trial was used as an ex adjuvantibus diagnosis, respondents heterogeneously reported using different doses of the drug. In BAD studies, cholestyramine was generally started at a low doses of 2 to 4 g/day and titrated based on response (maximum, 4‒24 g/day) [16]. When cholestyramine trial duration was considered, the majority of interviewed gastroenterologists reported a trial of 28 days, while other physicians reported shorter durations. Heterogeneous duration of cholestyramine trial from 4 to 12 weeks have been published [22]. However, lack of response to cholestyramine does not constitute per se exclusion of BAD and there is very little evidence to determine the relative role of 75SeHCAT testing versus using an empiric trial of BAST to make a diagnosis of BAD [22]. Therefore, in most guidelines, other factors were considered when making a recommendation for or against a diagnosis based on a cholestyramine trial [6, 16]. Despite evidence supporting the efficacy of bile acid sequestrants, treatment prescription remains suboptimal. Barriers such as perceived poor tolerability, side effects such as bloating or constipation, and uncertainties regarding dose optimization may deter clinicians from initiating therapy [12]. Patient-related concerns, including palatability and long-term adherence, may further complicate therapeutic decision-making [23].

About one out of five respondents among gastroenterologists and those with the 75SeHCAT availability believed that 7αC4 or FGF19 are accurate enough for BAD diagnosis. However, although these tests may have a good specificity for identifying patients with moderate BAD (75SeHCAT < 10%), they have insufficient sensitivity as diagnostic tests to be used alone and they are not widely available [24].

Notably, no difference was found in the reported satisfaction among physicians regarding therapies available for BAD, with a rate of satisfaction of about 31–34%. Furthermore, all respondents reported efficacy of therapies for BAD of less than 30%. Indeed, besides the high rates of remissions reported for BASTs in observational studies [2, 25], the few placebo-controlled trials available did not show that colestyramine or colesevelam were superior to placebo regarding bowel movements, although secondary non-clinical outcomes improved [15, 26–28]. However, the response to cholestyramine may vary according to the BAD etiology and its degree of severity. A metanalysis published in 2009 reported that in the pooled data from 15 studies there was a correlation between the severity of malabsorption and response to BAST: response to colestyramine occurred in 96% of patients with < 5% retention, 80% at < 10% retention and 70% at < 15% retention [2]. Long-term efficacy of BASTs may be hampered by several factors, such as uncertainty on the optimal dose to be used, different individual responses, side effects such as bloating and constipation, vitamin and concomitant drug malabsorption [23]. Colesevelam seems to gain some therapeutic response in BAD in patients nonrespondent to cholestyramine according to a trial published in 2015, where colestyramine was unsuccessful in 44% of cases, 47% of which responded to colesevelam [29]. Recently, a diagnostic and therapeutic RCTs assessing C4 accuracy vs 75SeHCAT and the therapeutic response to colesevelam found that colesevelam was superior to placebo at inducing remission of BAD diagnosed with C4 concentration greater than 46 ng/mL [30]. Secondary outcome data suggested similar efficacy in treating 75SeHCAT-defined BAD [30].

Finally, we found that more than half of the participants were not satisfied with their own knowledge of BAD and this gap significantly increased when 75SeHCAT test was not available in the place of origin. Almost all participants reported the need for updates on this condition. These findings suggest a critical need for targeted educational initiatives and broader access to diagnostic tools to reduce disparities in care [16, 22].

Our study has some limitations: first, the sample size was rather low leading to a possible error type II when performing comparison among groups, although the response rate was satisfying. Second, the overall knowledge of BAD may have been positively influenced by a selection bias, since most gastroenterologists and other physicians participating in the meeting expressed an interest in gastroenterological diseases. Also, it was not possible to use validated questionnaires or to use questions with pre-validated scales in order to explore BAD knowledge and management, thus influencing the reliability of our questions. Furthermore, some heterogeneity in our results may be influenced by the different training and clinical experience. However, our survey also has several strengths: it is the first report describing the knowledge and awareness of BAD in Italy and more generally in a European country among practicing physicians. These data may be used in the future to improve the awareness and knowledge of this condition, e.g. with focused updating courses. Moreover, we provided real-life data regarding the current epidemiology and management of patients with BAD in Italy according to tests and drugs availability, and its burden on healthcare resources.

In conclusion, BAD is a common condition with a multifaceted etiology. At least one-third of patients with IBS suffer from this condition. 75SeHCAT testing is the gold standard method for the diagnosis of BAD, although available only in a few Italian centers. 75SeHCAT availability influences the awareness and knowledge of this disease, possibly leading to a faster diagnosis and consequently reducing the burden of this disease for the patient and healthcare facilities. Therapies currently available for the treatment of BAD are often not able to guarantee adequate symptom relief. Updates on BAD are needed to fill in this knowledge gap, especially in geographic areas where 75SeHCAT is not available. 

## Data Availability

The datasets generated during and/or analysed during the current study are available from the corresponding author on reasonable request.
